# Unlocking the power of global collaboration: Building a stronger evidence ecosystem together

**DOI:** 10.1002/cl2.1401

**Published:** 2024-04-16

**Authors:** Zoe Jordan, Vivian Welch, Karla Soares‐Weiser

**Affiliations:** ^1^ JBI, Faculty of Health and Medical Sciences The University of Adelaide Adelaide South Australia Australia; ^2^ Campbell Collaboration Ottawa Ontario Canada; ^3^ Cochrane Collaboration London UK

Across the global evidence ecosystem, numerous organizations share a common vision and mission: to promote evidence‐based decision‐making worldwide. These organizations, including JBI, the Cochrane Collaboration, and the Campbell Collaboration, have each made an indelible imprint on the evidence‐based movement and have been identified as “a crucial mechanism to facilitate the synthesis, transfer, and implementation of evidence into health care policy and practice” (Pilla et al., 2022, p. 211). While the benefits of global collaboration have been well established for some time, achieving impact at scale will require a fundamental shift in mindset.

The COVID‐19 pandemic marked a turning point for evidence‐based health care and decision‐making. It provided a unique context whereby policymakers, health care providers, researchers, and the public required immediate access to trustworthy evidence to make decisions. We collectively faced major challenges in translating a rapidly evolving body of new evidence into tangible response efforts, with health policy decisions receiving unprecedented public attention. The “stress test” of COVID‐19, and the many post‐pandemic initiatives that followed, highlighted the need for more effective strategies, institutional mechanisms, and capacities to systematically mobilize and contextualize the best available evidence for rapid decision‐making for effective and equitable public health responses (Global Commission on Evidence to Address Societal Challenges, 2022; Stibbe & Prescott, 2022; World Health Organization Evidence‐informed Policy Network [EVIPNet], 2021).

Each of our organizations responded to COVID‐19 in different ways and were able to provide access to reliable evidence. Yet, it is essential to acknowledge the challenges of sustaining funding, upholding methodological rigor, and ensuring diversity and inclusivity in our collective endeavors. Our demonstrated success in enhancing global health care, education, and social policy underscores the value of collaborative, evidence‐based approaches in addressing the world's most pressing challenges.

We find ourselves at a unique juncture where our respective global collaborative evidence networks (JBI, Cochrane, and Campbell) must reimagine the way we work together to facilitate and engage in multidisciplinary, transdisciplinary, and interdisciplinary research, dissemination, knowledge sharing, and knowledge translation to generate impact at scale across the evidence ecosystem. It is time to develop interagency collaboration as a coherent program rather than a series of standalone efforts. There is significant potential in our ability to orchestrate, integrate, coordinate, and align our activities to identify opportunities for mutual benefit, learning, and impact.

## 1 A CALL TO ACTION

One of the most significant benefits of our respective global networks is our capacity to transcend geographic boundaries. By facilitating better global interagency collaboration, we enable the pooling of expertise and knowledge in the field of evidence‐based practice, and the result is a more holistic and nuanced understanding of complex issues, leading to improved decision‐making at both local and global levels. Examples of this may include much deeper collaboration on methodologies and standards for synthesis that reflect the diversity of evidence to respond to global challenges; a more coordinated approach to the prioritization of synthesis efforts to avoid duplication of effort; and better, more meaningful partnership on the contextualization or localization of evidence for policy and practice.

Going forward, it is incumbent upon us to support and strengthen our networks and the relationships between them, recognizing the invaluable contributions we can collectively make to improving the well‐being of individuals and communities around the world. The path to a brighter, more evidence‐based future lies in continued collaboration and our unwavering commitment to the delivery of trustworthy evidence. Collaboration *across* our global networks, not just within them, is now not merely a choice but a necessity in our increasingly interconnected world.
